# Integrating Mindfulness into the Subject of Physical Education—An Opportunity for the Development of Students’ Mental Health

**DOI:** 10.3390/healthcare10122551

**Published:** 2022-12-16

**Authors:** Roberto Delgado-Montoro, Alberto Ferriz-Valero, Olalla García-Taibo, Salvador Baena-Morales

**Affiliations:** 1 Department of General and Specific Didactics, Faculty of Education, University of Alicante, 03690 Alicante, Spain; 2 Department of Physical Education and Sport, Pontifical University of Comillas, CESAG-Mallorca, 07013 Palma de Mallorca, Spain; 3 Faculty of Education, Valencian International University (VIU), 46002 Valencia, Spain

**Keywords:** health, physical education, mindfulness, attention, sustainability

## Abstract

Stress, uncertainty, and the abuse of technologies are components that have a negative impact on the physical, social, and psychological health of young people. One of the aims of the Education for Sustainable Development (ESD) is to empower individuals to reflect on their actions, and mindfulness arises as one tool with an important potential to contribute on this matter. Therefore, the objective of this study was to assess the effects of mindfulness practices on the ability of students to focus their attention on external, internal or kinesthetic factors, awareness in acting, and acceptance. Consequently, a quasi-experimental study was developed to compare groups between the pre and post condition. The study participants were a total of 127 students (52 women) from 4th year of secondary school and 1st year of a achelor’s degree (16.5 ± 1.5 years). The sample was assigned by academic convenience, with 54 students in the experimental group and 73 in the control group. The intervention was carried out for 4 weeks. During this period, the experimental group participated in mindfulness activities such as guided meditations at the end of the PE session or challenges that stimulated the student in daily actions. The control group continued with the planned programming in physical education class. These groups were subjected to the following test: (1) Mindfulness for School Scale (MSS) and (2) Child and Adolescent Mindfulness Measure (CAMM). To analyze the results, the normality of the sample was evaluated through the Mann–Whitney U test, resulting as non-parametric. The search for possible differences between the groups was carried out by using the Wilcoxon test. The statistics showed that the experimental group presented significant improvements (*p* ≤ 0.05) in most of the measured parameters: external attention, kinesthetics attention, and mean of the CAMM. These results seem to show that the use of mindfulness could be an appropriate tool to be implemented in the school context in order to directly contribute to the mental health of high school students, and thus to an education for the sustainable development.

## 1. Introduction

### 1.1. The Current Lifestyle and Its Problems for the Youngest

Nowadays, people’s lifestyles are characterized by the presence of unhealthy behaviors, such as sedentary habits, due to the increased use of motorized transport and screens for work, education, and leisure time. The prevalence of physical inactivity and sedentary lifestyles has increased in recent years, standing at 28% of adults and 81% of adolescents globally [1]. Physical inactivity, in addition to being the fourth leading cause of premature mortality worldwide [2], can be one of the risk factors for the development of more than 35 chronic diseases and disorders [3], including obesity and overweight, which in turn rank as the fifth main cause of mortality worldwide. In addition to the consequences generated by physical inactivity at a physical level, it also has a negative impact at a psychological and social level. Moreover, the use of cell phones has not stopped growing in recent years [4], generating a social transformation and breaking with traditional interaction and socialization mechanisms [5]. This change has negatively impacted the social relations among young people, causing a deficit in communication skills; phobia; social isolation [6,7], deterioration of relationships with family members, teachers, partners, and among peers; worsening of academic and work performance [8]; and increasing depression and anxiety [9]. Many preadolescents show high levels of anxiety and/or behavioral problems that, without meeting certain clinical diagnostic criteria, imply restrictions on their daily lives [10]. This is closely related to technology addiction problems [11] and proof of this is that in the last decade the diagnoses of attention deficit hyperactivity disorder have increased by 30%, with nearly 250,000 Spanish minors taking psychostimulants to deal with this disorder [12]. Recently, a study that assessed the preoccupation and fears about the issues of the 21st century, such us climate changes, natural devastation, COVID-19, and war, showed that young people reported less psychological well-being than older adults, in terms of stress, anxiety, and depression [13]. Given this situation, the need to promote emotional and social competence and children’s well-being is especially important during the transition from childhood to adolescence [14].

### 1.2. Education for Sustainable Development and Mindfulness

Faced with this current multidimensional problem, which affects us environmentally, physically, psychologically, and socially, the United Nations seeks to reverse this reality and contribute to the care of people in general, and young people in particular, through the design and establishment of the Sustainable Development Goals (SDGs), detailed in 169 specific targets. Therefore, the collaboration of institutions on the pursuit of these achievements is essential [15]. Among them, the educational context is a key factor to collaborate with the achievement of these European goals, especially in those ages in which education is compulsory. In this regard, UNESCO proposed the term Education for Sustainable Development (ESD), with the mission to empower students to make responsible decisions towards the achievement of a just society with economic and environmental integrity, both in the present and in future generations [16]. Education for Sustainable Development (ESD) aims to develop competencies that enable individuals to reflect on their actions, considering their current and future social, cultural, economic, and environmental impacts from a local and global perspective. Considering the deeply rooted origin of human cultures and behaviors, there is a need to implement new strategies that allow to change, in a profound way, some of their worldviews, values, and beliefs [17]. In this direction, mindfulness meets certain characteristics that allow us to contribute to the achievement of ESD. In recent years, this type of practice has been introduced in various sectors of society, particularly in education, showing several benefits, as will be approached below. Mindfulness is defined as a person’s ability to focus attention on events, experiences, and states of the present moment, both external and internal [11]. Mindfulness refers to the state of bringing non-judgmental awareness to the present moment and implies two components: (1) self-regulation of attention and (2) orientating an individual to the present moment with curiosity, openness, and acceptance [18]. In other words, mindfulness represents a successful tool for promoting awareness, cultivating the ability to be attentive, and increasing subjective wellbeing. Therefore, the benefits generated in mindfulness facilitate emotional competence such as emotional intelligence, because of the ability to be aware of one’s own emotions, the feelings of others, or to feel empathy, in line with the goals of ESD. Kabat-Zinn [19] rightly stated, “We do not know what specific knowledge our children will need ten or twenty years from now, because the world, and their work when they come to it, will be so different from ours. What we do know, is that they will need to know how to pay attention, how to focus and concentrate, how to listen and learn, and how to be in wise relationship with themselves—including their thoughts and emotions—and with others”.

This change in the present scenario has attracted the attention and interest of psychologists, educationists, and researchers to the practice of mindfulness-based interventions with students, both with children and adolescents, or even with teachers, for enhancing their overall well-being. Most of the research conducted on mindfulness has focused on studying the impact of mindfulness practices on students. According to [20,21], different studies have shown that children and adolescents who practice mindfulness can experience improvements in three basic areas: in general well-being, in cognitive aspects, and in social and emotional skills. All of these aspects can be beneficial to them at some point in their lives when facing challenges and changes in their personal, biological, psychological, and psychological development. That is, mindfulness offers useful preparation beyond the classroom, as it provides people with important tools and strategies that could apply in a variety of settings throughout life [22].

Regarding the benefits and improvements in student well-being, different studies have shown that mindfulness helps to promote greater resilience to daily stress [22]. Children and adolescents are especially vulnerable to the harmful effects of toxic stress, which can cause attention problems, mood swings, emotional disturbances, sleep problems, and learning disabilities [23]. Therefore, reducing stress and anxiety levels has benefits both at a personal and academic level. In addition, this practice has been shown to be helpful in the treatment of depression in adolescence [24], as well as with symptoms of post-traumatic stress in children and adolescents [25]. In addition to reducing students’ stress levels, it improves their attention and concentration, so that mindfulness could improve the learning process of students, thus raising their academic performance and, consequently, their results [22]. Regarding emotional intelligence, there is evidence that this type of practice improves the ability to manage emotions in children and adolescents, contributing to the development of emotional self-regulation skills [26] and fostering empathy and compassion with peers [27]. Precisely, emotional self-regulation and social interaction appears as new content within mental health in the last educational curriculum for the physical education subject in Spain (Real Decreto 217/2022).

### 1.3. Mindfulness in the Physical Education Curriculum

A review study on research that implemented mindfulness in PE classes concluded that these are scarce [28]. However, the analysis of the secondary physical education curriculum (Real Decreto 217/2022) suggests a close relationship between this subject and mindfulness. This newest approach for the physical education subject in Spain sets out a holistic perspective that highlights physical, psychological, social, and environmental aspects. Students will have to learn how to manage their emotions and social skills. In this regard, it will be necessary to integrate cognitive and motor skills, but also affective-motivational and interpersonal relationships. In terms of health contents, the “Active and healthy life” block addresses three components of health (physical, mental, and social); this means developing postural education, relaxation, and positive relationships among others, in order to achieve a healthy lifestyle. 

In order to integrate healthy routines and responsible motor practice, developing the specific competence of “emotional self-regulation and social interaction” is needed. This means, “develop the processes aimed at regulating their emotional response to situations arising from the practice of physical activity and sport, and, on the other hand, developing social skills and promoting inclusive and constructive relationships. It is also emphasized the positive attitude to face challenges, regulating impulsivity, tolerating frustration and persevering in the face of difficulties. It involves the identification of emotions and feelings that are experienced within the motor practice, the positive expression of these and their proper management in order to constructively buffer the effects of unpleasant emotions and feelings that they generate, as well as to promote pleasant emotions. Likewise, in relation to one’s own body, it involves the development of skills for the preservation and care of personal integrity”. Regarding the collective level, the PE subject must specifically work on “putting into play social skills to face the interaction with the people with whom one converges in the motor practice. It involves dialoguing, debating, contrasting ideas and agreeing to resolve situations; expressing proposals, thoughts and emotions; listening actively, and acting assertively”. In fact, one of the basic skills refers to reflection on negative attitudes towards physical activity derived from preconceived ideas, prejudices, stereotypes, or negative experiences. Having presented the characteristics and benefits of mindfulness and the particularities of the EF curriculum, there is no doubt that there is a close relationship between both and that meditation is an ideal tool to implement in the classroom. However, more studies on mindfulness MF in secondary education curriculum planning are needed to evaluate its benefits [29]. Thus, the objective of this study was to assess the effects of mindfulness practices on the ability of students to focus their attention on external, internal or kinesthetic factors, awareness in acting, and acceptance.

## 2. Materials and Methods

### 2.1. Design

The methodological design of the study was quasi-experimental with quantitative pre- and post-test measures. Two different groups were formed in the different courses of the 4th year of secondary education and 1st year of bachelor’s degree. Only one of the two groups practiced activities focused on mindfulness in order to observe the differences obtained between the experimental group compared to the control group. The primary outcome measures were external, internal and kinesthetic attention, and mindfulness. Convenience sampling, a type of non-probabilistic or non-random sampling, was carried out. This type of sampling is valid when it includes members of the target population who meet certain practical criteria, such as easy accessibility, geographical proximity, availability at a given time, or willingness to participate in the study. This type of sampling is valid and common in quantitative studies, although the results obtained cannot be extrapolated to the overall study population [30]. In this research, the reference population would be adolescents. However, it should be noted that in a convenience sample, neither biases nor their probabilities are quantified [31]. Consequently, researchers can guarantee the extent to which participants will represent the population in terms of traits or research mechanism.

### 2.2. Participants

The total sample consisted of 127 students (52 females) from a high school in Alicante (Spain). The age range in the study was 16.5 ± 1.5. The experimental group (EG) was composed of 54 students of which 23 were girls and 31 were boys. The control group (CG) consisted of 73 individuals, 29 girls and 42 boys. In order to select the sample, inclusion criteria were assigned: no previous experience in meditation, being students at the school and agreeing to participate in the study. Exclusion criteria were: (1) having specific needs: e.g., autism or Down’s syndrome, (2) students with learning difficulties in reading comprehension of the target language, and (3) students with physical incapacity to perform the practical classes

### 2.3. Intervention

The mindfulness training was carried out during 3 school weeks in the second and third trimester of the 2021/2022 school year. The pre-test evaluation was conducted 1 week before the start of the training and the post-test evaluation was conducted one week after the end of the intervention. The mindfulness program was implemented in a total of six consecutive PE sessions, two classes per week. In these sessions, a 10-min guided meditation was conducted by the person in charge of this study during the calm-down phase. This consisted of an exercise of relaxation and awareness, in which one had to pay attention to the breath and body, as well as to thoughts without judgment, while visualizing situations (spaces and sounds of nature) that lead to a state of presence and calm (see https://youtu.be/f44boj9hQRk (accessed on 5 May 2022)). The instructors who conducted the sessions had previous experience as facilitators and were trained in mindfulness. In addition, a series of challenges were also developed to keep them in touch with mindfulness during the Easter holidays, activities that stimulated the student into aware daily actions. These challenges consisted of: Day (1): Mindful eating; day (2): Mindful shower; day (3): Three conscious stretches while breathing with awareness; day (4): Mindful walk; day (5): 7-min guided meditation (focused on awareness of breathing, bodily sensations, thoughts, and feelings) (see https://www.youtube.com/watch?v=XXXaoKi7IY0 (accessed on 5 May 2022)). All students completed the intervention. Only one student did not attend one session due to sickness and then followed the rest of the sessions normally.

**Figure 1:**
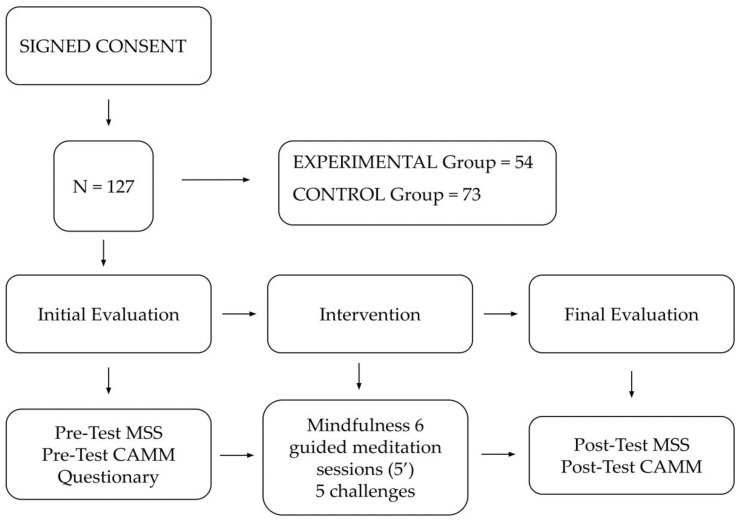
Intervention process.

The collaboration and permission of the school and PE teachers was requested. All participants were informed of the aims of the study and signed an informed consent for the release of data for scientific use. The ethical aspects presented in the Declaration of Helsinki were respected in the design of the study. This research was approved by the ethics committee of the University of Alicante with code UA-2022-03-17. It was emphasized to the participants that the information would be totally confidential, treated statistically, and that neither the center nor the parents would be informed about the results of the questionnaires of each student. In order to carry out the study, a letter of application was sent indicating the outline of the project, with the objectives, relevance of the research, methodology, sample inclusion/exclusion criteria, risks and benefits, commitment to comply with data protection legislation, commitment to confidentiality and anonymity, information sheet, informed consent, and annexes with the tests or surveys to be taken or description of the tests to be carried out. The aforementioned scheme can be observed in Figure 1, which shows the number of participants and the processes they underwent.

### 2.4. Instruments

The MSS, which measures levels of mindfulness in the school environment, and the CAMM Questionnaire, which measures the level of mindfulness, were used as assessment instruments.

#### 2.4.1. MSS (Mindfulness at School Scale)

The MSS [29] is a Likert scale whose score consists of an ordinal quantitative variable (1 = never to 5 = always). In this case, the 12 items of the questionnaire are written in a positive sense, so the result is the sum of the scale scores. The scale has an internal consistency of α = 0.84 and a test-retest stability of 0.78. The MSS measures three factors which the authors describes and labels as follows:

- Kinesthetic attention: referring to the subject’s ability to become aware of their movement and motor actions (items 5 and 12) (α = 0.74).
- External attention: referring to the subject’s ability to direct attentional resources towards events happening outside, attention to observation (items 1, 2, 3, 4, 6, 7, and 8). (α = 0.60).
- Internal attention: referring to the subject’s ability to direct attentional resources towards events occurring inwardly, attention to introspection (items 9, 10, and 11) (α = 0.66).

#### 2.4.2. CAMM (Child and Adolescent Mindfulness Measure)

The CAMM [32] is an assessment instrument composed of 10 items that measures acceptance and mindfulness with a Likert scale where the score consists of an ordinal quantitative variable (1 = never to 5 = always). In this case, the items of the questionnaire are written in a negative sense, so to obtain the final result the sum of the total score will be inverted. This questionnaire has mainly been used with children and adolescents aged 9–18 years. The test-retest reliability for the entire test according to the test authors is α = 0.88. The factor structure, internal consistency, and construct validity of the individuals were tested. Although this instrument has a one-dimensional factor structure, there are measure items regarding two aspects of mindfulness: acting with awareness (e.g., “It’s hard for me to pay attention to only one thing at a time”) and accepting without judgment (“I get upset with myself for having certain thoughts”) [33].

### 2.5. Statistical Analysis

According to [33], the statistical power of the sample size was calculated using the free software G*Power (See 3.1.9.6, University of Dusseldorf, Düsseldorf, Germany). The sample size, 54 participants and 73 participants per group, with an estimated medium effect size (0.5), and a significance of 95%, resulted in a power of 0.96. All continuous variables collected during the study were subjected to a normality test, specifically the Kolmogorov–Smirnov test. The data were also subjected to univariate statistical analysis for non-parametric samples, specifically the Mann–Whitney U and Wilcoxon test, to assess the differences between the experimental group (EG) and control group (CG) twice: pre- and post-intervention. The significance level was set at *p* < 0.05 in all cases.

A repeated-measures analysis of variance (ANOVA 2 × 2) mixed model was used when pre and post differences were identified, to give robustness to the analysis. The dependent variables were four: CAMM test, internal attention, external attention, and kinesthetic attention. Time (pre- and post-intervention) was the within-subject factor, whereas the group (control vs. experimental) was the between-subject factor. Levene test was used to check for homoscedasticity, the Mauchly test for sphericity, and the Box’s test for the equivalence of covariance matrices. All the assumptions were correctly met in the dataset. The effect size of the ANOVA was calculated by the partial eta-squared (η^2^_p_). The statistical programs used for the statistical analysis were Statistics Product and Service Solutions (IBM^®^ SPSS^®^ Statistics Version 24.0.0.0) (International Business Machines Corp., Madrid, Spain) and Microsoft Excel^®^ in its 2016 version (Microsoft Corp., Redmond, WD, USA). 

## 3. Results

### 3.1. Descriptive Statistics and Baseline Differences

Table 1 shows the descriptive statistics for the CAMM and MSS tests for the control and experimental groups at pre- and post-test.

At pre-test, both groups (EG vs. CG) presented similar starting values regarding the four research variables: external attention (Z = −0.814; *p* = 0.416), internal attention (Z = −0.745; *p* = 0.456), kinesthetic attention (Z = −0.849; *p* = 0.396) and CAMM test (Z = −1.377; *p* = 0.168).

### 3.2. Intra-Group Differences (Pre vs. Post-Test)

The Wilcoxon Test is shown in Table 2. This table will give information about the differences between the variables of each group (intra-group, pre vs. post-test). It can be observed that the EG shows significant differences in all study variables, except for internal attention that shows only a trend towards change. These variables show a positive change, i.e., to a higher score, which means that participants score higher in the post-test than in the pre-test. The CG shows a significant change in two variables (external and internal attention) and a trend towards change (Kinesthetic attention). To the contrary, in the CG, this significant change is negative, i.e., participants score smaller values in the post-test compared to pre-test.

### 3.3. Final Intergroup Differences

Post-test, both groups (EG vs. CG) presented different final values regarding the four research variables: external attention (Z = −2.427; *p* = 0.015), internal attention (Z = −3.281; *p* = 0.001), kinesthetic attention (Z = −1.960; *p* = 0.050), and CAMM test (Z = −6.050; *p* = <0.001). This means that the EG obtained higher values in all study variables.

### 3.4. Repeated-Measures Analysis of Variance Mixed Model (Two.Factor ANOVA)

Firstly, in regard to the CAMM, an interaction effect (Time × Treatment) was found (F(1) 17.463, *p* = <0.001; η^2^_p_ = 0.178). That is, the mindfulness implementation in PE significantly increased CAMM items compared to the control group. Secondly, according to the internal attention, an interaction effect (Time × Treatment) was found (F(1) 5.266, *p* = <0.022; η^2^_p_ = 0.041). That is, the mindfulness implementation in PE significantly increased internal attention compared to the control group. Finally, referring to the external and kinesthetic attention, no interaction effect (Time × Treatment) was found. That is, the mindfulness implementation in PE did not increase these variables significantly compared to the control group.

## 4. Discussion

The present study aimed to analyze how mindfulness influences the levels of external, internal, and kinesthetic attention; awareness in acting; and acceptance of adolescents. Considering the importance of the current moment in society as well as the curricular approach, which highlights the need to develop emotional self-regulation, mindfulness is a tool that could contribute to this purpose. However, few studies have developed these techniques in the PE classroom. This is the reason why the present study consisted of a mindfulness program integrated during the calm-down of the PE class as well as outside school hours, by daily mindfulness challenges to be implemented at home. After the end of the intervention, the data obtained showed very significant progress in the students in most of the variables analyzed, with a large number of positive ranges in the experimental group compared to the control group. On the other hand, no significant differences were observed between the groups in the case of external and kinesthetic attention.

The results from this study are aligned with other research, such us the study by [34], which showed how the practice of yoga (including mindfulness practice) in PE classes, based on different body postures, breathing exercises, relaxation, and meditation, contributed to numerous benefits at a physical, psychological, and social levels. In this sense, [35] also showed improvements in the levels of attention and state of calm after performing a mindfulness intervention with elementary school students. Moreover, there is the study by [36] that showed that introducing mindfulness in the school setting in Chile significantly decreased anxious and depressive symptomatology. In line with these results, although the present study did not include specific indicators of anxiety or depression, improving aspects such as introspection, acting with awareness, acceptance without judgment, and verbalization of your experiences, are deeply related to the emotional response. In a similar way, the benefits observed in this study may contribute to improved academic performance. In fact, [37] stated that including emotional training in the education curriculum is required since it is associated with higher academic results and a better transition to the next level of studies.

In order to increase the impact of the obtained results, increasing the study sample would be interesting. In fact, in the study by [38], they pointed out that enlarging the sample of mindfulness studies in education is necessary in order to verify the efficacy of this practice and generalize the obtained results. Furthermore, these authors mentioned that only 10% of the studies conducted with samples of university students had a control group; therefore, a positive aspect of the present article is that it compares the experimental condition with the control, which reinforces the strength of the results. On the other hand, although the study by [39], conducted with more than 400 high school students, concluded that there were no differences between men and women in overall relaxation habits, it would have been interesting to expand the detail by the comparison between genders. Moreover, analyzing the long-term impact of these interventions through longitudinal studies would be enriching, since the permanence of the achieved improvements over time has not been studied [40] and, therefore. it is not clear that a real change in lifestyle can be obtained. Nevertheless, it is a very encouraging outcome that short practices of only 10 min resulted in such good results. Moreover, the procedure presented facilitates the implementation of mindfulness in the PE classroom. After conducting this study, the response received by the participating students was very remarkable, since the vast majority showed absolute gratitude for having had the opportunity to carry out the proposed activities. Although some mindfulness activities can be easily implemented, in order to increase the success of the interventions, the training of teachers is very important [28]. In fact, after analyzing the PE curriculum, especially the latest update, teachers will require new tools according to the new content blocks to achieve the objectives, since the approach goes beyond the motor element. The PE teacher must update his or her training towards these new holistic trends in PE, which emphasizes emotional and social competencies. The PE curriculum demands student development and improving emotional self-regulation skills becomes essential. Among these new tools, mindfulness has been shown to be a successful and easily implementable option. Therefore, training teachers in mindfulness is required, first of all by applying it to themselves and their own lives, so that they can teach others in their educational context [41]. In line with the statement of these authors, the nature of these practices requires experimentation before applying it with students.

The subject of PE is not limited to the development of physical aspects, but also offers the possibility of working on psychological aspects. In fact, several studies showed that the practice of physical exercise reduces negative feelings and accentuates positive dimensions of psychological health [42]. In addition, PE provides the opportunity to work on interpersonal skills and decision making, aspects that will lead to an improvement of students’ emotional intelligence [43]. Therefore, body practices in PE should enhance the stimulation of body sensations such as tension, relaxation, pleasure, fatigue, suffering, overload, etc., using methods that promote awareness of the body through internalization practices such as meditation, yoga, and taijiquan [44]. In this regard, as [45] states, developing self-knowledge is essential to train students capable of participating in a social transformation, which requires focusing education towards personal and social development. It is necessary to promote methodological procedures that develop the awareness of the participants, both students and teachers, that is, to integrate the cognitive, affective, moral, and spiritual dimensions [46]. To contribute in this regard, the PE subject could integrate activities towards reflection or meditation, which can be perfectly implemented into the calm-down part of the sessions. Accordingly, in this research, the mindfulness activities were included in the final section of the sessions, without affecting the corresponding main content.

### Limitations and Future Proposals

As for the limitations and complications generated during the study and intervention, we can highlight certain aspects that were observed in the development of the activities. Firstly, the lack of mindfulness experience of the participants is a factor that could have adversely affected the experience if the students were not able to understand the questions in the questionnaires, since they are not used to this type of vision or activities. Therefore, making an introduction on this subject to both the control and experimental groups could have helped with this. Therefore, it is essential that mindfulness interventions are adapted to the different educational levels [28]. On the other hand, alternative tools could have been applied as those included by other studies such as [24], in which qualitative methods were used, consisted of collecting students’ experiences through personal reflections in diaries, interviews, reflection groups, etc. In addition to these limitations, it must be acknowledged that this research involved convenience sampling. This sampling means that the results obtained cannot be extrapolated to the general population studied.

Besides, better control of the practice conditions would have been necessary, since during the experience of this intervention, we observed difficulties for students in focusing on the practice, because initially they were not able to maintain silence and concentrate on the activity, and there were situations of laughs and distractions among them. For this reason, it would have been essential to emphasize the familiarization with this type of activities to teach the importance and value of these practices, so that the students take them seriously. For this purpose, performing these tasks in a large space is relevant, guaranteeing distance among the participants, with dim lighting, preventing other people from observing the practice, aspects that guarantee certain intimacy. Besides, using appropriate sound equipment that facilitates audio monitoring helps the students to focus their attention on the activity instructions. In terms of strengths, the daily challenge proposal has been a very motivating component as it has made students aware of the multitude of ways in which mindfulness can be easily practiced and applied.

Finally, compared to other countries, research in Spain on implementing mindfulness in the PE class is practically missing, especially at the infant, primary, and secondary levels [28]. According to the education for sustainable development, further mindfulness interventions are necessary to contribute to understanding the benefits mental well-being can have on education, particularly to the subject of PE due to its curricular affinity. 

## 5. Conclusions

In conclusion, the different issues addressed in this article related to the current lifestyle of the youngest are associated with negative psychological well-being, and the education for sustainable development aims to solve this situation by developing competencies of self-regulation. Mindfulness arises as a practice that consists of training awareness, which could enhance students’ mental health. This study showed improvements in the ability of students to focus their attention on external, internal or kinesthetic factors, awareness in acting, and acceptance. These variables contribute to emotional self-regulation and improved mental health. Overall, this study justifies and supports the implementation of mindfulness practices in the physical education classes in line with the goals of the education for sustainable development. This is particularly relevant considering the topicality of the issue and the lack of related studies. Future research should focus on implementing actions that can contribute to the mental health of the students within the physical education curriculum, in line with the demands of the current education on sustainable development in facing the challenges of today’s world.

## Figures and Tables

**Table 1:** Descriptive statistics.

		Pre-Intervention				Post-Intervention			
			Mindfulness at School Scale				Mindfulness at School Scale		
		CAMM	EA ^1^	IA ^2^	KA ^3^	CAMM	EA ^1^	IA ^2^	KA ^3^
Control (*n* = 73)	Mean	1.86	3.24	3.79	3.37	1.88	3.18	3.69	3.32
	Standard error	0.07	0.07	0.10	0.10	0.07	0.07	0.09	0.10
	Median	1.90	3.14	3.66	3.50	1.90	3.14	3.66	3.50
	Standard deviation	0.62	0.67	0.85	0.87	0.60	0.62	0.78	0.86
	Range	2.90	3.14	3.00	3.50	3.20	2.71	3.00	3.50
Experimental (*n* = 54)	Mean	2.05	3.11	3.92	3.24	2.74	3.47	4.15	3.61
	Standard error	0.08	0.09	0.09	0.11	0.10	0.10	0.09	0.08
	Median	2.00	3.14	4.00	3.50	2.70	3.57	4.33	4.00
	Standard deviation	0.59	0.71	0.70	0.81	0.78	0.77	0.69	0.64
	Range	2.30	3.14	2.66	3.50	3.80	3.14	3.00	2.50

^1^ External Attention, ^2^ Internal Attention, ^3^ Kinesthetic Attention.

**Table 2:** Wilcoxon test (intra-group differences, pre- vs. post-test).

		Experimental Group (EG)					Control Group (CG)				
		Positive	Negative	Ties	Z	Sig.	Positive	Negative	Ties	Z	Sig.
CAMM		43	9	2	4.602	<0.001	31	22	20	1.407	0.159
Mindfulness at School Scale	EA ^1^	36	15	3	2.056	0.040	8	21	44	−2.698	0.007
	I A ^2^	28	14	12	1.755	0.079	2	13	58	−2.883	0.004
	KA ^3^	30	15	9	2.766	0.006	2	8	63	−1.941	0.052

^1^ External Attention, ^2^ Internal Attention, ^3^ Kinesthetic Attention.

## Data Availability

Not applicable.
